# Bibliographical Notices

**Published:** 1840-04-18

**Authors:** 


### BIBLIOGRAPHICAL NOTICES.

*Dictionnaire de Medicine, or Repertoire General des Sciences Médicale, &c. Deuxime Edition.* Paris, 1839.

Volumes nineteen and twenty of the Dictionary of Medicine have now appeared, and bring the work much nearer its completion. The last number contains the articles to the heading *Ner*, but leaves some important articles for the few volumes which remain to complete the set. The work is incomparably the best dictionary extant, and is of a very convenient size, large enough to contain full monographs on all the important subjects, yet much more convenient for reference than the voluminous Dictionary of the Medical Sciences. The comparatively large demand for this work in America, sixteen or seventeen copies being received by a single importing bookseller at Philadelphia, is a sufficient proof that the work is properly appreciated in the United States.

The two volumes contain numerous important articles. The nineteenth includes one on the mammæ, on the history of medicine, on meningitis, menstruation, mercury, and menorrhagia. The twentieth contains the anatomy and diseases of the spinal marrow, an article on glanders both of the human subject and the horse, and a very elaborate series of articles on the nerves and the nervous system. Of most of these articles we have only to speak in terms of unqualified praise; but of the article on meningitis, by M. Guersent, we regret hat we should have reason to complain. The present state of our knowledge in reference to this subject is very satisfactorily set forth, but the mode in which that knowledge has been attained is imperfectly stated. As we shall probably return to this matter on another occasion, we need not enter into it more fully at present.

Still, the dictionary, as a whole, is a monument which will long endure and be a lasting testimony of the ability, patient industry, and clear logical description of the authors. In general, the diffuseness of style which is so objectionable in many French writers is completely avoided, and the subjects are described in the shortest and most concise manner possible.

---

*An Inquiry concerning the diseases and functions of the Brain, the Spinal Cord, and the Nerves.* By Amariah Brigham, M. D. New York: George Adlard, 1840. 12mo., pp. 327.

Dr. Brigham's work is written with the praiseworthy object of inducing the numerous physicians of the United States to unite in the study of the diseases of the brain. That is, by exciting the simultaneous labours of a large proportion of the physicians of this country, he thinks much may be done to clear up this very interesting department of pathology. But we will let the author speak for himself.

"The object of the following work is to call the attention of those practitioners of medicine into whose hands it falls, to the importance of the nervous system; and to persuade them to embrace every opportunity that is presented, for studying its functions and diseases.

"For this purpose, I have endeavoured to give a partial summary of what is now known respecting this system. I have collected a large number of cases explanatory of its diseases and functions—cases that are scattered through many volumes, to which I have added a considerable number that have fallen under my own observation; and have thus sought to indicate the way that this system should be studied, in order to increase our knowledge of its functions, and our means of remedying its diseases.

"In the second part, I have briefly treated of a number of diseases, the pathology of which is not yet settled. I have not sought to give full accounts of these, but to direct attention to a few important circumstances and such as require further investigation.

"Affections of the nervous system have always existed, and always formed a considerable proportion of the diseases, and those the most obscure, by which the human system has been assailed.

"But they have vastly increased with the increase of civilization, and now constitute a far greater proportion of the diseases of mankind, than in past ages, and consequently demand far more attention. If this volume induces but a few to investigate the affections of this system with care, to record and make known the facts they observe and thus add something to our knowledge of its functions and diseases, it will be all that the author expects."

That there is much to be done in this matter, is evident enough; not only should physicians, as a matter of duty to the profession and to humanity, pay greater attention to the diseases of the brain, but as a matter of comfort to themselves. There would then be less disposition to overlook them in their forming stage, and to class many important organic alterations of the brain under the vague title of affection of the stomach, &c. This is, in fact, very often confounding a mere accidental developing cause with the real mischief, which often quietly proceeds for a long time almost unnoticed.

Dr. Brigham gives a good account of the leading organic diseases of the brain. He has connected this portion of his work with a large mass of cases collected from the later authorities upon the diseases of the brain, although he has not quite brought the subject down to the present day. In fact, his reading seems to have been much more extensive amongst the writers of the first twenty years of the present century, than with those that are more recent.

This is apparent from the imperfect account which he has given of the acute hydrocephalus of children; a disease said to be upon the increase. Dr. Brigham refers it to many causes, and, amongst others, lays great stress upon the premature and excessive cultivation of the intelligence. This, however, is evidently a fallacy; in no class of children is there a more absolute lack of intellectual culture, even of the humblest order than amongst the children of the poorer classes at Paris, and yet hydrocephalus is, as far as we can learn from observation, more frequent there than in other countries. The truth is, that Dr. Brigham has not gone back to the usual course of the disease, that is, a tuberculous deposit, or, at least, a tuberculous diathesis.

The chapters on chorea and hysteria are admitted by the author to be less elaborate than those of the first part of the work. The remark of Dr. Serres that he found an alteration of the tuberculi quadrigemini in one case of chorea, is quoted in the work. Admitting the fact to be correct, the case is quite isolated, and we are sorry to see that it is occasionally cited as a fact of some importance. It is, in reality, just as unconnected with true chorea, as would be a tubercle in the lungs or bronchial glands. It is now perfectly understood that there is no organic lesion in chorea.

